# Polymyoclonus aggravated by neck flexion as the isolated presenting symptom of Hirayama disease: case report

**DOI:** 10.1186/s12883-020-01904-z

**Published:** 2020-09-01

**Authors:** Jun-Young Kim, Su-Keong Hwang, Soonhak Kwon, Jin-Sung Park

**Affiliations:** 1 Department of Orthopedic Surgery, School of Medicine, Catholic University of Daegu, Deagu, South Korea; 2 Department of Pediatric Neurology, School of Medicine, Kyungpook National University Children’s Hospital, Daegu, South Korea; 3 Department of Neurology, School of Medicine, Kyungpook National University, Kyungpook National University Chilgok Hospital, 807 Hoguk-ro, Daegu, South Korea

**Keywords:** Polymyoclonus, Hirayama disease, Flexion, Aggravation, Case report

## Abstract

**Background:**

We report a rare case of an 18-year-old male with unilateral hand tremor who was finally diagnosed with Hirayama disease (HD).

**Case presentation:**

An 18-year-old male presented with unilateral polymyoclonus that aggravated with neck flexion. The patient did not complain of muscle weakness or muscle atrophy. The needle electromyography showed giant motor unit potentials in right cervical 7 and 8 innervated muscles. The cervical magnetic resonance imaging on supine and flexion state showed prominent forward effacement of posterior dural sac that was compatible with HD.

**Conclusions:**

HD usually presents with unilateral distal hand weakness, muscle atrophy and tremor. Although it is a benign and self-limiting disease, early diagnosis may lead to less clinical deterioration. Moreover, electromyography should be completed in the differentiation of young male patients who present with polymyoclonus without hand weakness or atrophy.

## Background

Benign focal amyotrophy, or Hirayama disease (HD), is a rare self-limiting cervical myelopathy that predominantly affects adolescent males. It is clinically characterized by asymmetric weakness of the upper extremity muscles that are innervated by C7-T1 roots. Recent literature describes the high prevalence of cold paresis, fasciculation, paresthesia, numbness, and tremor in association with the disease [1, 2]. Only recent studies have recognized the presence of tremor in HD; it has been clinically described in terms of jerky, irregular, tremulous movements; myoclonic tremor; and polymyoclonus [3–5]. We report a unique HD patient, who presented with polymyoclonus as the initial symptom.

## Case presentation

**Fig. 1:**
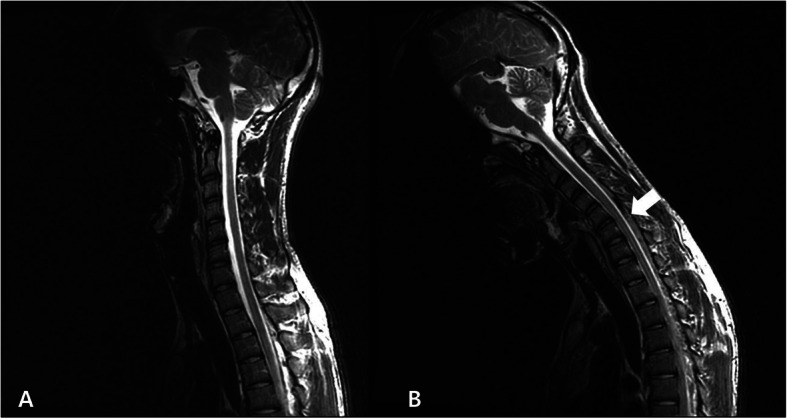
Cervical magnetic resonance images captured with the patient in the supine position (**a**) were unremarkable, while when the patient was in the flexed position, anterior effacement of the posterior dural sac was observed with venous engorgement at the C6–8 levels (**b**)

An 18-year-old boy presented with a 1-year history of hand tremor of the right hand, which worsened with neck flexion. He had no previous history of autoimmune diseases, including thyroid disease or family history of genetic disease. The initial neurological examination revealed no significant muscle weakness or atrophy, and except for a mildly decreased right triceps tendon reflex, deep tendon reflexes were normal. He complained of hand tremor of the right hand that worsened during neck flexion (Video 1). The hand tremor was irregular and jerky myoclonic tremors that were confined to his right hand, which was compatible with polymyoclonus. The routine laboratory findings, including complete blood count, electrolytes, liver function, renal function, thyroid function, and autoimmune-related tests, were all within normal limits. Electroencephalography and nerve conduction findings were unremarkable. However, needle electromyography revealed unit potentials in muscles (abductor pollicis brevis, first dorsal interosseous and triceps muscles) innervated by the C7 to T1 roots that were of significantly higher amplitude than other muscles. With a working diagnosis of HD, we ordered cervical magnetic resonance imaging (MRI) in both neutral and flexed positions, which revealed a clear anterior displacement of the posterior dural sac compressing the C7–8 roots (Fig. 1), which is an imaging hallmark of HD. With the above clinical, electrophysiological, and imaging findings, the patient was diagnosed with HD.

## Discussion and conclusions

The pathogenic mechanism of HD is yet to be elucidated, but recent imaging studies have strongly suggested microcirculatory ischemia in the anterior horn cells caused by direct cord compression resulting from forward effacement of the posterior dural sac [6]. This can be easily observed using cervical MRI in the flexed posture, which shows direct compression of the C7-T1 levels, corresponding with the muscle weakness. Another hypothesis postulates that the pathogenesis of HD can be explained by an imbalance between growth of the vertebral column and the spinal canal [1]. It is noteworthy that a large cohort study revealed a 90% male preponderance, which indirectly reflects the important role of accelerated vertical growth during puberty. Besides weakness, cold paresis is known to be commonly observed among HD patients, as is tremor, which has a reported prevalence of approximately 70–80% among HD patients in contemporary studies [1, 2].

The underlying mechanism of hand tremor has not yet been elucidated, but a recent report of multiparametric brain MRI on an HD patient revealed brain hyperactivation that may explain the hand tremor associated with HD. A case report described coexisting juvenile myoclonic epilepsy in a young male HD patient [5]. Another hypothesis is the muscle myoclonus that originate from the motor nerve injury cause by neuroexcitatory mechanism [7]. Whatever is the cause, it is important to recognize HD-associated tremors, and narrowing down the differential diagnosis is pivotal for arriving at the appropriate treatment. Most publications that describe the clinical features of HD describe a high prevalence of hand tremor or polymyoclonus among the comorbid symptoms, but tremor as a predominant clinical symptom of HD is not well described.

Although HD is a self-limiting disease, it is important to recognize the disease that should lead to the avoidance of neck flexion because it usually aggravates the symptoms. Few reports have described positive results after cervical surgery or cervical collar therapy that reduced functional disability among HD patients [8, 9].

Only recent publications have begun to illustrate and emphasize polymyoclonus as a clinical feature of HD. A recent case report described a 17-year-old boy who presented with polymyoclonus along with decreased muscle bulk associated with weakness. Of note, the myoclonus was aggravated by outstretching the arms [3]. Another report described a 20-year-old male with asymmetric muscle weakness and myoclonic tremors in resting and arm-outstretched postures [5]. These reports emphasized the importance of suspecting HD when a patient presents with predominant polymyoclonus along with mild muscle weakness.

Our report is of clinical significance as it describes the first reported patient who presented with isolated polymyoclonus without muscle weakness or muscle atrophy. The patient also had no complaints related to muscle weakness or muscle atrophy. Furthermore, the myoclonus was aggravated by neck flexion, corresponding well with the hypothesis of direct cord compression, which is generally accepted as the main pathophysiologic mechanism of HD. Clinicians should be aware of a self-limiting disease, such as HD, when evaluating young,most frequently male patients who present with predominant functional polymyoclonus that is worse with neck flexion and perform an electromyography study for the earlier diagnosis that may prevent further clinical deterioration.

## Supplementary information

**Additional file 1: Video 1.** The video shows polymyoclonus of the right hand which is aggravated by neck flexion.

## Data Availability

N/A

## Abbreviation

HD: Hirayama disease
